# An Ensemble Classifier for Ordinal Outcomes in High‐Dimensional Genomics Data

**DOI:** 10.1002/pst.70097

**Published:** 2026-05-10

**Authors:** Heranga K. Rathnasekara, Sinjini Sikdar

**Affiliations:** ^1^ Department of Mathematics and Statistics Old Dominion University Norfolk Virginia USA

**Keywords:** bagging, classification, ensemble, genomic data, high‐dimensional, ordinal

## Abstract

Analysis of genomics data for predicting disease outcomes is a fast‐growing field in medical research. There often exist categorical, specifically, ordinal outcomes that need to be predicted based on genomic profiles. This has led to recent development of some high‐dimensional ordinal classification methods that can address the large dimensionality of the genomic covariate set. These high‐dimensional ordinal models tend to vary widely in their performance depending on the data they are applied to and the evaluation criteria used. In this article, we outline an ensemble ordinal classifier that integrates different ordinal modeling approaches through bootstrap‐based model evaluation, multi‐metric performance assessment, and rank aggregation to produce a final prediction that can alleviate the uncertainty of relying on a single model. Through multiple simulated studies and real genomic data analyses, we show that the ensemble method consistently ranks among the top‐performing models. These findings underscore the potential of ensemble learning to improve the robustness and predictive accuracy of high‐dimensional ordinal classification in genomic research.

## 1 Introduction

Analysis of omics data has become popular in medicine and health sciences as it can lead to better understanding of a disease mechanism, improved treatment outcomes, as well as accurate prediction of possible risk of a disease [1, 2]. Due to several hundreds or thousands of gene expressions being analyzed simultaneously, the data generated in genomic disease studies are often high‐dimensional in nature. Most of the statistical models that have been developed to analyze these high‐dimensional genomics data focused on either continuous (e.g., biomarker levels) or binary (e.g., presence or absence of a condition) outcomes. However, in genomic studies, disease outcomes can be categorical with natural ordering (ordinal) [3]. For example, cancer can be classified into different stages that reflect its progression, level of pain can be categorized as low, medium and severe based on its intensity, severity of stroke can be classified with the Modified Rankin Scale, traumatic brain injury severity can be categorized using the Glasgow Outcome Scale [4], and so on. While the methods for continuous outcomes are not applicable to the ordinal outcomes, combining multiple ordered categories into a binary outcome for modelling purposes can severely impact the power of study due to loss of information [5, 6].

Despite being less explored than their continuous counterparts, in the recent past, a few methods have been developed for modeling ordinal outcomes in high‐dimensional data that can be applied to genomic studies. These are mostly penalized likelihood‐based approaches for modeling the association between the gene expressions and some function of the ordinal outcomes. These include penalized models based on cumulative logit or probit functions [7, 8], penalized models based on a continuation ratio function [9], and penalized models based on adjacent ordinal categories [10]. Moreover, a penalized model based on a Bayesian framework has also been proposed that allows different types of prior distribution assumptions for the proportion of important genes [11]. Recently, a random forest–based method for ordinal outcomes has been proposed which can be used for both low‐ and high‐dimensional datasets as an alternative to penalized models [12]. Random forests, unlike the previously mentioned regression‐based penalized models, are based on decision trees and allow for non‐linear relationships. Other alternatives to penalized models include support vector machines and neural networks [13, 14, 15]. While each of these methods has its own advantages and disadvantages, their performance varies widely depending on the dataset and the evaluation criteria used. Generally, there is no consensus on any individual classification method being the ‘best’ with respect to several evaluation criteria and different data types. In order to overcome or alleviate this uncertainty over choosing a single best method, in recent years, it has become highly desirable to use an ensemble learning approach, combining predictions of several individual classification methods for improved performance [16, 17]. For example, an ensemble approach based on bagging and boosting has shown to provide more accurate results for ordinal classification compared to several individual classifiers in various transportation datasets [18]. Another ensemble approach for ordinal classification based on a genetic‐based methodology for a weighted voting system has led to improved results in minimizing classification error [19]. In addition to these, a study showed that relying on ordinal decision‐tree‐based ensemble approaches led to much better performance in classifying the daily COVID‐19 growth rate factor based on environmental factors and containment measures in several regions of Italy [20].

In the construction of an ensemble classifier or predictor, the relative contributions of individual models in the final prediction constitute an important factor. While most of the existing ensemble approaches use a single evaluation criterion for determining the relative contributions or the weights of the component high‐dimensional models, such ensembles can produce widely variable results if the evaluation metric is changed. To reduce this variability in the ensemble outputs, Datta et al. proposed an ensemble learning approach for classification in high‐dimensional studies, combining bagging and a rank aggregation technique [21]. This proposed classifier is flexible and highly adaptive in nature, having several advantages over the traditional ensemble approaches. Not only does it allow flexibility to produce good performance consistently on many different datasets, but it also considers a multi‐objective approach by evaluating a variety of performance measures simultaneously [21]. This approach of Datta et al. [21], despite showing better performance compared to any individual classification method across several simulated and real datasets, was restricted to models and evaluation metrics for binary classification problems only.

In this article, we extend this idea of adaptive and multi‐objective ensemble learning approach to the classification of ordinal outcomes in a high‐dimensional genomic study setting. We share the same flexibility and adaptability of the approach by combining bootstrap aggregation and rank aggregation in our ordinal classification problem. While building the ensemble model, we consider several state‐of‐the‐art individual ordinal models and high‐performance evaluation metrics. It is to be noted that, for simplicity and ease of use, only individual models with a working R software package are being considered in demonstrating our ensemble algorithm in this article. However, our method can also be applied by substituting any of the individual component high‐dimensional models with other suitable alternatives in the ensemble if needed.

The rest of the article is organized as follows. In Section 2, we describe the ensemble approach for classification of ordinal outcomes in a high‐dimensional setting. We also briefly describe the individual models and the evaluation metrics that are included in the ensemble. This section also contains a description of the rank aggregation technique utilized in the ensemble. In Section 3, we show the usefulness of the ensemble approach in several simulation settings and two different real‐data applications. Finally, we end this article with a discussion in Section 4.

## 2 Methods

### 2.1 The Algorithm

In this section, we provide a detailed description of the algorithm for the ensemble classifier [21], adapted for ordinal outcomes.

Let the training set X have n individuals, each having data on p features, such as genes. Let $y_{i}$ denote the ordinal class level for the ith individual in the training set, i=1,2,…,n. Suppose the testing set Z has m individuals, each with data on the p features. Our aim is to build a classifier using the training data {X,y} and predict the ordinal class level, say $\left\{y^{*}\right\},$ for the m individuals in the testing set.

The following are the steps for developing the ensemble classifier in a training set and its prediction of ordinal outcomes in a new testing set.

Step 1: Randomly draw a bootstrap sample of size n from the training set. The data for the individuals who are selected in the bootstrap sample are denoted as $\left\{X^{\text{boot}},y^{\text{boot}}\right\}$ and will be referred to as the ‘bootstrap training’ data. The data for the individuals who are not selected in the bootstrap sample will be referred to as the ‘out‐of‐bag’ data, $\left\{X^{\mathrm{oob}},y^{\mathrm{oob}}\right\}$.

Step 2: (a) Identify K models, say $M_{1},M_{2},\ldots ,M_{K}$, specifically designed to handle ordinal outcomes in high‐dimensional data. In particular, the following five models are considered in the ensemble: two different forms of penalized continuation ratio model (CR_L1 and CR_L1_path) [9, 22], a penalized cumulative logit model (CL_L1) [7], a cumulative logit model with generalized monotone incremental forward stagewise penalization method (CL_GMIFS) [8], and ordinal forest (OF) [12]. Details about each of these models are provided in Section 2.2.

(b) Train the K models on the ‘bootstrap training’ data, $\left\{X^{\text{boot}},y^{\text{boot}}\right\}$.

Step 3: (a) Identify V evaluation criteria, specifically designed for ordinal outcomes, to assess the performances of the K models. In this article, the following four evaluation criteria are considered: mean absolute error (MAE), Kendall's rank correlation coefficient, accuracy, and gamma statistic. Details about each of these evaluation measures are provided in Section 2.3.

(b) Using the trained models obtained in step 2, predict the ordinal class levels of the ‘out‐of‐bag’ samples, $\left\{{\hat{y}}^{\mathrm{oob}}\right\}$ based on each model. Assess the performance of each of the K trained models across the V evaluation criteria. Note that, the true class levels of these ‘out‐of‐bag’ samples, $\left\{y^{\mathrm{oob}}\right\}$, are already known.

(c) Rank the K models based on their performances across the V evaluation criteria. Let $E_{j}$ denote the ranked list of the K models based on the jth evaluation criteria, j=1,2,…,V.

Step 4: The V ranked lists obtained in step 3 are aggregated using a rank aggregation method [23], producing a single ranked list of the models with an overall best performance.

Step 5: Repeat steps 1–4 N times. At the end of this step, the ensemble will consist of N individual best performing classifiers, $M_{\left(1\right)}^{l},l=1,2,\ldots ,N$, based on N sets of bootstrap training and testing data.

Step 6: Using each of the N individual best performing classifiers, $M_{\left(1\right)}^{1},M_{\left(1\right)}^{2},\ldots ,M_{\left(1\right)}^{N}$, obtained in step 5, predict the outcome levels $\left\{y^{*}\right\}$, for the testing set Z. Then, use majority voting to determine the most frequent ordinal class level for each individual in the testing set, resulting in the final set of classifications. In the rare event of a tie in the voting counts, additional bootstrap iterations can be performed and the vote totals updated until the tie is resolved.

**FIGURE 1:**
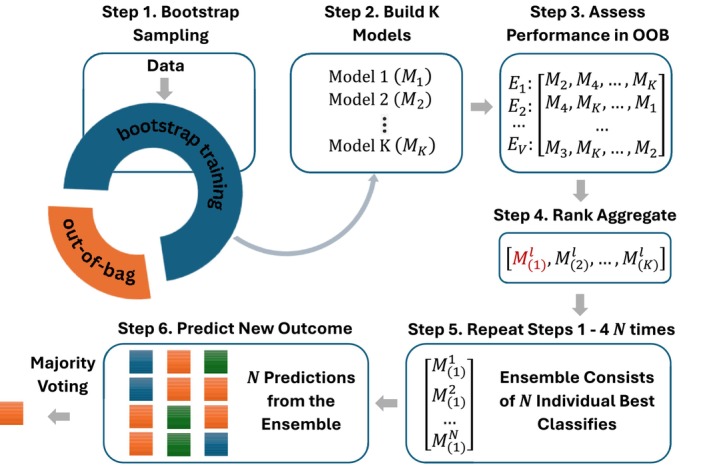
A flow diagram illustrating the steps of the ensemble algorithm (adapted from Datta et al. [21]).

A flow diagram describing all the steps for the construction of the ensemble classifier and its application for predicting the ordinal outcomes of new samples is shown in Figure 1.

### 2.2 Models for Ordinal Outcomes

In this section, we will discuss each of the individual models that are used in the construction of the ensemble classifier for ordinal outcomes in high‐dimensional data.

#### 2.2.1 Continuation Ratio Model With $L_{1}$ Norm Penalization

This model was proposed by Archer et al. to analyze ordinal outcome in high‐dimensional data [9]. Their approach combines $L_{1}$ norm penalization technique with a continuation ratio model. Although the continuation ratio method can be set up using different ways, we considered the backward formulation technique as it is commonly used when estimating the odds of higher ordinal outcome category compared to lower category. Here, we provide a brief description of the $L_{1}$ norm penalized continuation ratio model with backward formulation technique [9].

Suppose $y_{i}$ and $x_{i}$ denote the ordinal outcome class level and a p− dimensional vector of covariates, respectively, for individual i, i=1,2,…,n. Suppose there are a total of C ordinal class levels. For the *c*th ordinal class, c=1,2,…,C, the logit of the conditional probability is modelled as

$$\text{logit}\;\left(P\left(Y=c|Y\leq c,X=x\right)\right)=\log\left(\frac{P\left(Y=c|Y\leq c,X=x\right)}{P\left(Y<c|Y\leq c,X=x\right)}\right)=\alpha_{c}+\beta_{c}^{T}x$$

Assuming $y_{\mathrm{ic}}=1$ if the outcome is in category c and 0 otherwise, ensuring that each observation belongs to exactly one category, the likelihood for the continuation ratio model is given by

$$L\left(\beta |y,x\right)=\prod_{i=1}^{n}\left(\prod_{j=2}^{C}\delta_{j}^{y_{\mathrm{ij}}}{\left(1-\delta_{j}\right)}^{1-\sum_{c=j}^{C}y_{\mathrm{ic}}}\right)$$

where

$$\delta_{j}\left(x\right)=P\left(Y=j|Y\leq j,X=x\right)=\frac{\exp\;\left(\alpha_{j}+\beta_{j}^{T}x\right)}{1+\exp\;\left(\alpha_{j}+\beta_{j}^{T}x\right)}$$

Note that, the full parameter vector β is of length (C−1)(p+1). A constrained continuation ratio model is fitted where the original dataset is reconstructed by forming C−1 subsets, assuming a common set of slope parameters, $\left(\beta_{1},\ldots ,\beta_{p}\right)$. Further details about the reconstruction of the subsets can be found in Archer et al. [9].

The $L_{1}$ norm penalization [24] is introduced in the continuation ratio model as

$$\underset{\beta }{\text{argmax}}\left(L\left(\beta |y,x\right)-\lambda \sum_{m=1}^{p}|\beta_{m}|\right)$$

The final model is selected with the minimum AIC, and the estimated coefficients are used to obtain the predicted ordinal class level by taking the argmax of class probabilities. The R package, *glmnetcr*, is used for fitting this penalized continuation ratio model. From now on, we will refer to this model as CR_L1.

#### 2.2.2 Continuation Ratio Model With Path‐Wise $L_{1}$ Norm Penalization

The $L_{1}$ norm penalization, as described in the previous section, leads to an automatic variable selection effect [24]. Park and Hastie [22] modified the criterion of $L_{1}$ norm penalization with a regularization as

$$\hat{\beta }\left(\lambda \right)=\underset{\beta }{\text{argmax}}\left(L\left(\beta |y,x\right)-\lambda \sum_{m=1}^{p}|\beta_{m}|\right)$$

where a predictor–corrector method is implemented to determine the entire path of the coefficient estimates by varying λ. In other words, it determines $\hat{\beta }\left(\lambda \right)$ by varying λ,0<λ<∞. In particular, this method initiates with the largest λ for which $\hat{\beta }\left(\lambda \right)$ is non‐zero and computes a series of solution sets. At each step, it estimates the coefficients with a smaller λ based on the previous estimate. This regularization method is a smoother version of forward stepwise selection method, providing models throughout the entire range of complexity. Further details about this approach can be found in Park et al. [22].

This path‐wise penalization approach for ordinal outcomes is incorporated in the *glmpathcr* R package. We have considered a continuation ratio model with backward formulation technique, as described in the previous section. The parameters for the final model are selected using minimum AIC and predicted ordinal classes are assigned based on the argmax of the fitted class probabilities. From now on, this continuation ratio model with path‐wise $L_{1}$ norm penalization will be referred to as CR_L1_path.

#### 2.2.3 Cumulative Logit Model With $L_{1}$ Norm Penalization

For an ordinal outcome $y_{i}$ and a p− dimensional vector of covariates $x_{i}$, a cumulative logit model is defined as

$$\text{logit}\;\left(P\left(Y\leq c\;|\;X=x\right)\right)=\log\left(\frac{P\left(Y\leq c\;|\;X=x\right)}{1-P\left(Y\leq c\;|\;X=x\right)}\right)=\alpha_{c}+\beta^{T}x$$

c=1,2,…,C−1, i=1,2,…,n.

Assuming $y_{\mathrm{ic}}=1$ if the outcome is in category c and 0 otherwise, the likelihood for the cumulative logit model is given by

$$L\left(\beta |y,x\right)=\prod_{i=1}^{n}\left(\prod_{j=1}^{C}\delta_{j}^{y_{\mathrm{ij}}}\right)$$

where $\delta_{j}\left(x\right)=P\left(Y=j|X=x\right)$ can be obtained as P(Y≤j|X=x)−P(Y≤j−1|X=x). The $L_{1}$ norm penalization, as previously described, is introduced in the cumulative logit model. The final model is selected with the minimum AIC, and the predicted ordinal class level is assigned based on the argmax of the fitted class probabilities. Further details about this method can be found in Wurm et al. [7] The R package, *ordinalNet*, is used to fit this model. From now on, we will refer to this model as CL_L1.

#### 2.2.4 Cumulative Logit Model With Generalized Monotone Incremental Forward Stagewise Penalization

This method applies penalization on cumulative logit‐based likelihood similar to the previous approach while differing in the manner through which the penalization is implemented. The incremental forward stagewise (IFS) method applies penalization where the model coefficients are updated in small increments while enforcing monotonicity to the $L_{1}$ norm penalization [25]. This can often lead to a different solution path compared to the standard lasso penalty. However, the standard IFS method is not valid for categorical, hence ordinal, outcomes. Therefore, a generalized monotone incremental forward stagewise (GMIFS) model was introduced where a full solution path was constructed using IFS updates. Starting from all coefficients equal to zero, the algorithm iteratively updates the coefficient with the largest absolute gradient of the log‐likelihood, taking small incremental steps. The optimal model along this solution path was selected as the step that minimized the AIC. Archer et al. implemented the GMIFS method on several models for ordinal outcomes [8]. In this project, we have considered the previously described cumulative logit model with the GMIFS method, and predicted ordinal classes are assigned based on the argmax of the fitted class probabilities. Further details about this approach can be found in Archer et al. [8] The R package *ordinalgmifs* has been used to fit this model in our simulated and real data applications. From now on, we will refer to this method as CL_GMIFS.

#### 2.2.5 Ordinal Forest

This constituent model, used in our ensemble classifier, is different from all the other constituent models as it is the only model that does not use any form of penalty or shrinkage while addressing the high dimensionality problem. Rather, this method is based on the notion that a latent continuous outcome variable is underlying the observed ordinal outcome variable which can be used for prediction purposes. The Ordinal Forest is essentially a prediction method for ordinal outcomes, based on the approach of the popular random forest, that makes use of the out‐of‐bag error estimates in the construction of the forest [12]. Below is a brief description of this method:

Given a dataset with n samples, let $x_{i}$ denote the covariate vector of the ith sample, i=1,2,…,n. Suppose there are C ordinal classes where the outcome $y_{i}$, i=1,2,…,n, is the class value of the outcome, that is, each $y_{i}\in \left\{1,2,\ldots ,C\right\}$.

A heterogeneous collection of B score sets is obtained where each score set consists of C (transformed) midpoints of the C adjacent intervals forming a division of the interval [0, 1] into C adjacent intervals. Let $\left\{d_{1}^{b},d_{2}^{b},\ldots ,d_{C+1}^{b}\right\}$, denote the divisions of the bth set, b=1,2,…,B. The bth score set is created as $\left\{s_{1}^{b},s_{2}^{b},\ldots ,s_{C}^{b}\right\}$, where $s_{j}^{b}=\Phi^{-1}\left(c_{j}^{b}\right)$ and $c_{j}^{b}=\left(d_{j}^{b}+d_{j+1}^{b}\right)/2$, j=1,…,C, b=1,2,…,B. For the bth set, a continuous outcome variable is created as $\left\{z_{1}^{b},z_{2}^{b},\ldots ,z_{n}^{b}\right\}$, where each class value j in the ordinal outcome variable $\left\{y_{1},y_{2},\ldots ,y_{n}\right\}$ is replaced by the jth value in the score set $\left\{s_{1}^{b},s_{2}^{b},\ldots ,s_{J}^{b}\right\}$, j=1,…,C, b=1,2,…,B.

For the bth score set, a random forest is constructed using the continuous outcome variable $\left\{z_{1}^{b},z_{2}^{b},\ldots ,z_{n}^{b}\right\}$ with $N_{T}$ trees, and out‐of‐bag predictions are obtained. Using the out‐of‐bag predictions of the continuous outcome variable, predictions of the ordinal outcome variable are obtained, and a performance score is assigned to the random forest model. The performance score is assigned using a weighted sum of Youden's index across all classes [12]. For our applications, we have considered equal weights for each class in the Youden's index to ensure that each class is classified with the same accuracy [12]. Let I denotes the set of indices of the top 10 random forests with the best performance scores. Then, for each j∈{1,…,C+1}, the average of the $d_{j}^{b}$ values for which b∈I is computed, resulting in a final set of C+1 divisions denoted as $d_{1},\ldots ,d_{C+1}$. A new score set, $\left\{s_{1},\ldots ,s_{C}\right\}$, is created using the new division set $\left\{d_{1},\ldots ,d_{C+1}\right\}$. A new continuous outcome variable is created as $\left\{z_{1},\ldots ,z_{n}\right\}$ using the new score set $\left\{s_{1},\ldots ,s_{C}\right\}$, as described before. Finally, a random forest is fitted using $\left\{z_{1},\ldots ,z_{n}\right\}$ with $N_{F}$ trees, and predictions of the ordinal outcome are obtained by averaging class probabilities across all trees in the forest and assigning each observation to the class with the highest average probability. Further details about the application of this method and its usage in predicting an ordinal outcome can be found in Hornung [12]. The R package *ordinalForest* is used to fit this model with B=1000 heterogeneous score sets, $N_{T}=500$ trees per score set, and $N_{F}=5000$ trees in the final forest using the optimized score set in all our applications. From now on, we will refer to this method as OF.

### 2.3 Evaluation Metrics

In this section, we describe the metrics that are considered to assess the performances of the individual models in the ensemble method.

#### 2.3.1 Mean Absolute Error

The MAE measures the average of the absolute differences between the predicted and the true ordinal class labels. Assuming that there are C ordinal class labels, let $\left\{y_{1},\ldots ,y_{n}\right\}$ and $\left\{{\hat{y}}_{1},\ldots ,{\hat{y}}_{n}\right\}$ denote the true and the predicted class labels of n individuals in the data. Then, the MAE is defined as

$$\mathrm{MAE}=\frac{1}{n}\sum_{i=1}^{n}|y_{i}-{\hat{y}}_{i}|$$

For this calculation, the C ordinal classes were mapped to equally spaced integers 1,2,…,C, preserving the ordinal structure. This measure indicates the average deviation in predictions, with values ranging between 0 and C−1. A lower value of MAE indicates a better predictive performance of a method.

#### 2.3.2 Kendall's Rank Correlation Coefficient

The Kendall's rank correlation coefficient, introduced by Kendall, is a nonparametric measure of strength of association between two ordinal variables. In ordinal classification, this measure is often used to assess the agreement between the predicted and the true class labels. Accounting for ties, the Kendall's rank correlation coefficient [26] $\left(\tau_{b}\right)$ is defined as

$$\tau_{b}=\frac{A-B}{\sqrt{A+B+T_{t}}\sqrt{A+B+T_{p}}}$$

where A and B denote the number of concordant and discordant pairs, respectively, while $T_{t}$ and $T_{p}$ denote the number of ties in true class labels and predicted class labels, respectively. This measure ranges from −1 to +1, its value reflecting the degree of agreement between the two variables. A higher value of $\tau_{b}$ indicates a better agreement between the true and the predicted values, and, hence, stronger predictive performance of the underlying method.

#### 2.3.3 Accuracy

This measure calculates the proportion of correctly identified class labels, defined as follows:

$$\text{Accuracy}=\frac{1}{n}\sum_{i=1}^{n}I\left(y_{i}={\hat{y}}_{i}\right)$$

This measure provides an evaluation of classification accuracy, and its value ranges from 0 (worst) to 1 (best).

#### 2.3.4 Gamma Statistic

The gamma statistic, denoted as γ, is another metric measuring the degree of association between two ordinal variables [27]. The γ statistic is defined as

$$\gamma =\frac{A-B}{A+B}$$

where A and B denote the number of concordant and discordant pairs, respectively. The γ ranges from −1 to +1. Higher the value of γ, stronger is the predictive performance of a method.

### 2.4 Rank Aggregation

Rank aggregation is a method of combining multiple ordered lists into a single ordered list in a meaningful way. Since different evaluation metrics, described in the previous section, can lead to widely different ordered lists of prediction methods for the same data set, it becomes essential to combine these lists into a single comprehensive ranked list through rank aggregation. In our ensemble method, we have considered the rank aggregation technique, introduced by Pihur et al. [23] Here, we provide a brief description of the technique.

Assuming that $E_{j}$ denote the ordered list of the K models based on the jth evaluation criteria, j=1,2,…,V, the objective function is defined as

$$\Psi \left(\eta \right)=\sum_{j=1}^{V}d\left(\eta ,E_{j}\right)$$

where η denotes any ordered list of the K models, and d denotes the distance function measuring the similarity between the ordered lists η and $E_{j}$, j=1,2,…,V. The objective function is minimized using the cross‐entropy Monte‐Carlo algorithm to find that η which would minimize the total distance between η and $E_{j}$ s. For metrics where lower values indicate better performance, such as MAE, the model with the lowest value is assigned rank 1, whereas for the other metrics, the model with the highest value gets rank 1. We have considered Spearman's footrule distance as a distance measure [28]. The R package *RankAggreg* is used for the rank aggregation [29].

## 3 Results

In this section, we describe the application of the ensemble method for ordinal outcome classification in various simulated datasets as well as in real genomic disease studies.

### 3.1 Simulation Studies

We simulated continuous gene expression datasets to evaluate the performance of the ensemble classifier in classifying an ordinal outcome. We considered three different simulation scenarios as discussed below:

#### 3.1.1 Simulation Scenario 1

In this simulation scenario, we considered a data generation process that was first introduced in Fu et al. [30] We considered n=300 samples each having expression levels for p=1000 genes. The predictor matrix, say $X_{n\times p}$, was generated from a multivariate normal distribution with mean 0 and variance–covariance matrix Σ, where $\Sigma =\rho^{H}$. Here, H represents the absolute difference matrix of indices, with the (*i*, *j*)th element defined as $\rho^{|i-j|}$, i,j=1,…,1000. A moderate correlation structure was assumed among all the predictors with ρ=0.5.

Using the predictor matrix $X_{n\times p}$, a latent continuous outcome variable, $Y^{\prime }$, was generated as

$$Y^{\prime }=X\beta +\varepsilon$$

where *ε* ∼ logistic(0,1) and $\beta_{j}\in \left\{\pm 0.7\right\}$, j=1,…,1000. The latent continuous outcome, $Y^{\prime }$, was transformed into an ordinal outcome variable, Y, using percentiles as thresholds. In our study, we assumed three ordinal class levels by categorizing $Y^{\prime }$ with its 33.3rd and 66.7th percentiles, resulting in 100 samples in each ordinal class.

After the data generation, we randomly split the data into 80% training data and the remaining 20% testing data. The ensemble method, as described in Section 2.1, was trained using the training data with N=100 bootstraps, while the testing data was used for final predictions. We evaluated the performance of the ensemble method using the four evaluation criteria, as described in Section 2.3. For comparison, we also trained the individual models that were used within the ensemble, separately on the same training data and predicted the outcomes in the testing data. The results were averaged over 100 Monte‐Carlo iterations.

Table 1 shows the performance of the ensemble method and that of the constituent models. In this dataset, the ensemble method has the lowest MAE, and the highest values of $\tau_{b}$, accuracy and γ, indicating strong overall performance across the evaluation metrics. Among the individual models, CL_GMIFS has the lowest MAE and the highest $\tau_{b}$, CL_L1 has the highest accuracy, and OF has the highest value of γ. The performances of CL_L1, CL_GMIFS, and CR_L1_path are very close across the evaluation metrics. While OF has the highest γ and performs similarly on $\tau_{b}$ and accuracy, its MAE is relatively high. The CR_L1 method has weaker performance with lowest values of $\tau_{b}$, accuracy, and γ. Overall, the ensemble method shows robust and balanced performance across all metrics. Figure S1 shows boxplots summarizing the distribution of the performance measures for all methods across the 100 Monte‐Carlo iterations. The interquartile ranges (IQRs) are generally similar across all methods.

**TABLE 1:** Performances of the ensemble method and the individual algorithms when all predictors are associated with the ordinal outcome and are correlated among themselves.

Model	MAE	$\tau_{b}$	Accuracy	γ
CR_L1	0.6938 (0.1167)	0.2120 (0.1528)	0.4067 (0.0833)	0.3261 (0.2346)
CR_L1_path	0.6773 (0.1042)	0.2307 (0.1475)	0.4128 (0.0833)	0.3566 (0.2237)
CL_L1	0.6708 (0.1000)	0.2362 (0.1069)	0.4187 (0.0708)	0.3662 (0.1805)
CL_GMIFS	0.6648 (0.1167)	0.2389 (0.1398)	0.4170 (0.1000)	0.3750 (0.2181)
OF	0.8357 (0.1417)	0.2319 (0.1653)	0.4140 (0.1000)	0.4058 (0.2747)
Ensemble	**0.6378** (0.1167)	**0.2564** (0.1272)	**0.4229** (0.0667)	**0.4763** (0.2311)

*Note:* Interquartile ranges (IQRs), based on 100 Monte‐Carlo iterations, are reported in parentheses. Bold values indicate the best performance for each metric.

Abbreviations: CL_GMIFS: cumulative logit model with generalized monotone incremental forward stagewise penalization; CL_L1: cumulative logit model with $L_{1}$ norm penalization; CR_L1: continuation ratio model with $L_{1}$ norm penalization; CR_L1_path: continuation ratio model with $L_{1}$ norm penalization and path‐wise approach; OF: ordinal forest.

#### 3.1.2 Simulation Scenario 2

In the previous simulation scenario, we assumed that all the p=1000 predictors were associated with the outcome. In this scenario, we considered a variation by assuming that only the first 10 predictors were associated with the outcome. Thus, we considered $\beta_{j}\in \left\{\pm 0.7\right\}$ for j=1,…,10 and $\beta_{j}=0$ for j=11,…,1000. All other parameter choices remained the same as in simulation scenario 1.

Table 2 shows the simulation results of the ensemble method and the individual algorithms for this scenario. In this dataset, the ensemble method has the lowest MAE and the highest γ, indicating strong performance on these two evaluation metrics. The CR_L1 method, which was the weakest performer in the previous scenario, has the highest values of $\tau_{b}$ and accuracy, performing better than the ensemble method on these two metrics. Overall, both the ensemble and the CR_L1 methods demonstrate competitive performance in this scenario. Although CR_L1_path has slightly higher accuracy than the ensemble method, its performance is weaker on the other three evaluation metrics. In this scenario, CL_L1 shows relatively weaker overall performance, with the lowest values of $\tau_{b}$, accuracy, and γ, and the second highest MAE. The OF method has the highest MAE and generally low values on the other three metrics. The CL_GMIFS method shows intermediate performance across all the metrics. Figure S2 shows boxplots summarizing the distribution of the performance measures for all methods across the 100 Monte‐Carlo iterations. While the CR_L1_path, CL_GMIFS, and OF methods show wider IQRs for certain evaluation metrics, the IQRs are generally similar across the other methods.

**TABLE 2:** Performances of the ensemble method and the individual algorithms when only the first 10 predictors are associated with the ordinal outcome and all predictors are correlated among themselves.

Model	MAE	$\tau_{b}$	Accuracy	γ
CR_L1	0.5743 (0.1641)	**0.4268** (0.2047)	**0.4966** (0.1600)	0.6013 (0.2482)
CR_L1_path	0.6007 (0.2095)	0.3649 (0.3053)	0.4797 (0.1833)	0.5300 (0.4131)
CL_L1	0.6345 (0.1677)	0.3068 (0.1889)	0.4412 (0.1154)	0.4530 (0.3010)
CL_GMIFS	0.6122 (0.2513)	0.3479 (0.2775)	0.4577 (0.1844)	0.5093 (0.4071)
OF	0.7502 (0.3017)	0.3311 (0.2723)	0.4554 (0.1333)	0.5360 (0.3805)
Ensemble	**0.5673** (0.1519)	0.4032 (0.2159)	0.4720 (0.1671)	**0.6349** (0.2347)

*Note:* Interquartile ranges (IQRs), based on 100 Monte‐Carlo iterations, are reported in parentheses. Bold values indicate the best performance for each metric.

Abbreviations: CL_GMIFS: cumulative logit model with generalized monotone incremental forward stagewise penalization; CL_L1: cumulative logit model with $L_{1}$ norm penalization; CR_L1: continuation ratio model with $L_{1}$ norm penalization; CR_L1_path: continuation ratio model with $L_{1}$ norm penalization and path‐wise approach; OF: ordinal forest.

We further evaluated the performance of the ensemble method in the presence of smaller and unequal sample sizes across the ordinal classes, similar to the Crohn's disease (CD) dataset described later in Section 3.2. The latent continuous outcome, $Y^{\prime }$, was transformed into an ordinal outcome variable, Y, by categorizing $Y^{\prime }$ using quantile‐based cut points chosen to match the class proportions observed in the CD dataset, resulting in ordinal classes with approximately 42, 26, and 59 observations, respectively. Table S1 shows the performance of the ensemble method and the individual constituent methods. In this scenario, the ensemble method has the highest $\tau_{b}$ and γ, whereas CR_L1 has the highest accuracy and CL_GMIFS has the lowest MAE. The MAE and accuracy of the ensemble method are very close to those of the top‐performing methods. Although the MAE values of all methods are generally higher than those in the previous scenario with 100 samples in each class, the ensemble method continues to show consistently balanced performance across all metrics under class imbalance and small sample sizes.

In an additional simulation scenario, we further examined the robustness of the ensemble method under departures from the proportional odds assumption while retaining the smaller and unequal class sizes described above. To introduce deviations from proportional odds, the ordinal outcome was generated using two latent continuous variables with different coefficient vectors. Specifically, two latent scores were generated as $Y_{1}^{\prime }=X\beta^{\left(1\right)}+\varepsilon_{1}$ and $Y_{2}^{\prime }=X\beta^{\left(2\right)}+\varepsilon_{2}$, where $\varepsilon_{1}$, $\varepsilon_{2}$ ∼ logistic(0,1) independently. As considered before, only the first 10 predictors were associated with the outcome with $\beta_{j}^{\left(1\right)},\beta_{j}^{\left(2\right)}\in \left\{\pm 0.7\right\}$ for j=1,…,10, while the remaining coefficients $\beta_{j}^{\left(1\right)},\beta_{j}^{\left(2\right)}=0$ for j=11,…,1000. The coefficient vectors $\beta^{\left(1\right)}$ and $\beta^{\left(2\right)}$ were generated such that they differ for at least some predictors, allowing the effects of predictors to vary across cumulative logits and thereby violating the proportional odds assumption. The ordinal response variable Y was then generated from the two latent scores using a sequential thresholding approach to produce approximately 42, 26, and 59 observations in the three ordinal classes. Table S2 shows the performance of the ensemble method and the individual constituent methods. The ensemble method has the best overall performance with the lowest MAE and the highest values of $\tau_{b}$, accuracy, and γ. Among the individual models, CR_L1 performs best with values only slightly worse than the ensemble method. Overall, these results show that the ensemble method maintains robust and consistently balanced performance even when the proportional odds assumption is violated.

#### 3.1.3 Simulation Scenario 3

In the previous simulation scenarios, we assumed that all the p=1000 predictors were correlated among themselves. As opposed to that, this simulation study was conducted to evaluate the performance of the ensemble method and the individual algorithms when the predictors are independent of each other. As in simulation scenario 2, we assumed that only the first 10 predictors were associated with the outcome. The predictor matrix, $X_{n\times p}$, was generated as follows. For the first 10 predictors, that is, for j=1,…,10,

$X_{\mathrm{ij}}∼N\left(1,1\right)$ for i=1,…,100,

$X_{\mathrm{ij}}∼N\left(3,1\right)$ for i=101,…,200, and

$X_{\mathrm{ij}}∼N\left(5,1\right)$ for i=201,…,300.

For the remaining predictors, that is, for j=11,…,1000, $X_{\mathrm{ij}}∼N\left(0,1\right)$ for i=1,…,300.

This generated predictor matrix ensured that only the first 10 predictors were differentially expressed between the three ordinal classes, similar to the simulation scenario 2.

The results of this simulation study are presented in Table 3. The CR_L1_path method has the best overall performance on this dataset, with the lowest MAE and the highest values of $\tau_{b}$ and accuracy. While the ensemble method does not outperform the CR_L1_path method on these three metrics, its performance is very similar. The OF method has the highest *γ* but performs relatively poorly in the other metrics, with the highest MAE and the lowest values of $\tau_{b}$ and accuracy. In this scenario, all the individual models except OF show similar patterns of performance, with generally high $\tau_{b}$ and accuracy and low MAE. Overall, *γ* values are similarly high across all methods. Notably, CR_L1, which was the top performing individual method in some of the previous scenarios, does not show a clear advantage over the other individual methods here. Overall, the ensemble method does not outperform any individual method in this scenario, but it maintains a performance that is consistently close to the top performing method. Figure S3 shows boxplots summarizing the distribution of the performance measures for all the methods across the 100 Monte‐Carlo iterations. The IQRs are very similar across all the methods.

**TABLE 3:** Performances of the ensemble method and the individual algorithms when only the first 10 predictors are associated with the ordinal outcome and the predictors are uncorrelated among themselves.

Model	MAE	$\tau_{b}$	Accuracy	γ
CR_L1	0.1865 (0.0667)	0.8234 (0.0760)	0.8150 (0.0667)	0.9692 (0.0298)
CR_L1_path	**0.1722** (0.0667)	**0.8350** (0.0652)	**0.8285** (0.0667)	0.9730 (0.0247)
CL_L1	0.1747 (0.0500)	0.8321 (0.0532)	0.8261 (0.0500)	0.9733 (0.0225)
CL_GMIFS	0.1783 (0.0667)	0.8283 (0.0570)	0.8223 (0.0667)	0.9718 (0.0238)
OF	0.3468 (0.0100)	0.7577 (0.0702)	0.6635 (0.0833)	**0.9798** (0.0340)
Ensemble	0.1747 (0.0833)	0.8337 (0.0656)	0.8263 (0.0708)	0.9754 (0.0253)

*Note:* Interquartile ranges (IQRs), based on 100 Monte‐Carlo iterations, are reported in parentheses. Bold values indicate the best performance for each metric.

Abbreviations: CL_GMIFS: cumulative logit model with generalized monotone incremental forward stagewise penalization; CL_L1: cumulative logit model with $L_{1}$ norm penalization; CR_L1: continuation ratio model with $L_{1}$ norm penalization; CR_L1_path: continuation ratio model with $L_{1}$ norm penalization and path‐wise approach; OF: ordinal forest.

### 3.2 Applications on Disease Studies

We evaluated the performance of the ensemble method on two genomic datasets, discussed in this section.

#### 3.2.1 An Application to a Breast Cancer Study

We analyzed a publicly available miRNA expression dataset (accession: GSE22216) [31], obtained from the Gene Expression Omnibus database [32]. This dataset was a part of a joint analysis of mRNA–miRNA involving 207 breast cancer patients. The tumor grades of the patients are divided into three ordinal categories. There were 42 patients with tumor grade value 1, 81 patients with tumor grade value 2, and 63 patients with tumor grade value 3, while the tumor grade of the remaining patients was missing. For our analysis, samples with missing tumor grades were excluded. This study included expressions for 735 miRNAs, measured using Illumina Human v1 MicroRNA expression beadchip.

**FIGURE 2:**
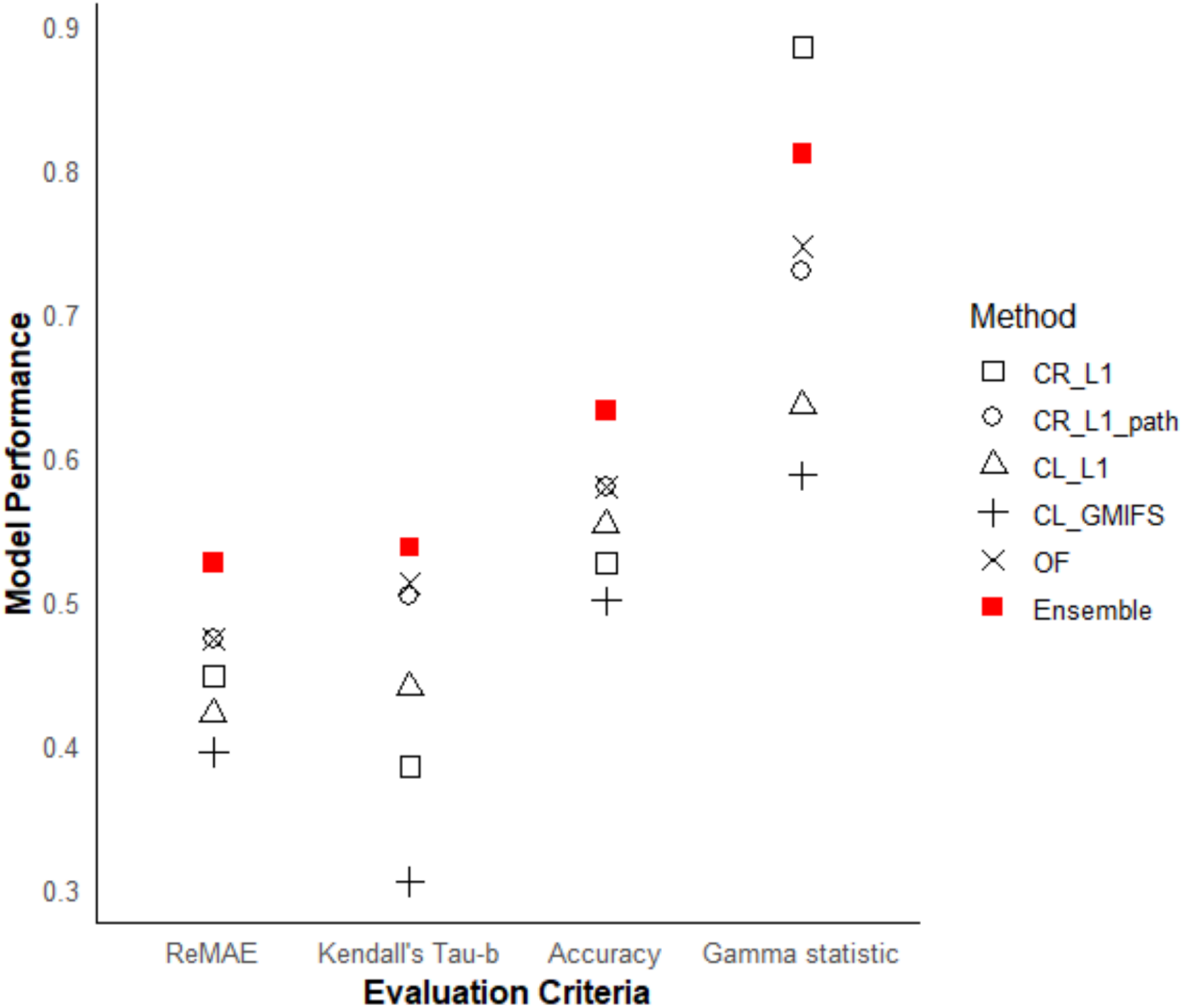
Performances of the ensemble method and the individual algorithms on the breast cancer study (GSE22216). The figure reports four evaluation measures: Reverse MAE (ReMAE), $\tau_{b}$, Accuracy, and γ. ReMAE is defined as the reversed mean absolute error (MAE), so that higher values indicate better performance, aligning its direction with the other metrics, for which higher values naturally indicate better performance. CL_GMIFS: cumulative logit model with generalized monotone incremental forward stagewise penalization; CL_L1: cumulative logit model with $L_{1}$ norm penalization; CR_L1: continuation ratio model with $L_{1}$ norm penalization; CR_L1_path: continuation ratio model with $L_{1}$ norm penalization and path‐wise approach; OF: ordinal forest.

We randomly split the data into 80% training and 20% testing sets. The training set was used to build the ensemble method, and the testing set was used for its evaluation. For evaluation of the method, we considered the four metrics, discussed in Section 2.3. For comparison, the individual algorithms were fit on the same training set and evaluated on the testing set. The performances of all the methods in the test set are shown in Figure 2. The ensemble method has the lowest MAE and the highest values of $\tau_{b}$ and accuracy among all methods. The γ value of the ensemble method is the second highest and lower than that of CR_L1. However, CR_L1 performs considerably worse than the ensemble method on the other three metrics. The performance of CL_GMIFS is relatively weaker with the highest MAE and the lowest values of $\tau_{b}$, accuracy, and γ. The CR_L1_path and OF methods perform similarly across all four metrics. Overall, these results indicate that the ensemble method provides balanced and consistently competitive performance compared to the individual methods in this cancer dataset.

#### 3.2.2 An Application to a Crohn's Disease Study

We analyzed a publicly available gene expression dataset (accession: GSE3365) [33], where the pre‐processed data was obtained from the Gene Expression Omnibus database [32]. This dataset included transcriptional profiles of peripheral blood mononuclear cells from 127 subjects, comprising of 42 healthy individuals, 26 patients with Ulcerative Colitis (UC), and 59 patients with CD. Expression levels for 22,284 probe sets were measured using the Affymetrix GeneChip HG‐U133A Array. In this study, we defined the ordinal class levels as normal <UC < CD [9].

The data was split into 80% training and 20% testing sets. The ensemble method and the individual algorithms were applied on the training set and evaluated on the testing set. The performances of the methods in the test data are shown in Table 4. In this study, both the ensemble method and CR_L1 have the lowest MAE, with the ensemble method having the highest accuracy and CR_L1 having the highest $\tau_{b}$ and γ. The CR_L1_path method has slightly higher $\tau_{b}$ than the ensemble method, but it has lower accuracy, higher MAE, and similar γ. The CR_L1_path and CL_L1 methods have identical MAE and accuracy values, but CL_L1 has considerably lower $\tau_{b}$ and slightly lower γ. The CL_GMIFS and the OF methods have the highest and identical MAE values, with OF having higher $\tau_{b}$, accuracy and slightly higher γ. Overall, CR_L1 performs best on several metrics, while the ensemble method shows competitive performance across all evaluation metrics.

**TABLE 4:** Performances of the ensemble method and the individual algorithms on the Crohn's disease study (GSE3365).

Method	MAE	$\tau_{b}$	Accuracy	γ
CR_L1	**0.3077**	**0.8218**	0.6923	**1.0000**
CR_L1_path	0.3462	0.7541	0.6538	0.9152
CL_L1	0.3462	0.6742	0.6538	0.8929
CL_GMIFS	0.3846	0.7140	0.6154	0.9053
OF	0.3846	0.7541	0.6538	0.9091
Ensemble	**0.3077**	0.7426	**0.7326**	0.9170

*Note:* Bold values indicate the best performance for each metric.

Abbreviations: CL_GMIFS: cumulative logit model with generalized monotone incremental forward stagewise penalization; CL_L1: cumulative logit model with $L_{1}$ norm penalization; CR_L1: continuation ratio model with $L_{1}$ norm penalization; CR_L1_path: continuation ratio model with $L_{1}$ norm penalization and path‐wise approach; OF: ordinal forest.

## 4 Discussion

Classification of ordinal outcomes in high dimensional data is a complex, but an increasingly important area of research. Several traditional methods for ordinal classification, originally developed for low‐dimensional datasets, have been adapted for handling the sparsity of a high dimensional dataset. Over the years, methods based on regularization, dimension reduction, deep learning, as well as tree‐based methods have shown to provide reasonably well performance in classifying ordinal outcomes in high‐dimensional data. However, the performance of a particular classification method is highly dependent on the evaluation criteria used. This dependency creates uncertainty, as it is often unclear which method will perform the ‘best’ for a given application. As a possible remedy to this problem, it might be wise to integrate several individual classification methods into an ensemble, which can harness the strengths of the individual methods and deliver a more robust and reliable solution. In this article, we have borrowed the idea of the multi‐objective ensemble classifier developed by Datta et al. [21], originally applied to binary outcome classifications and adapted it for classification of ordinal outcomes.

On evaluating the ensemble classifier and comparing it against its constituent individual methods across multiple simulation studies and genomic datasets on diseases, it showed that the ensemble classifier has a consistently strong and balanced performance, often outperforming or closely matching the best‐performing individual method across various evaluation criteria. Moreover, the ‘best’ performing individual method varies from one study to another for a given evaluation metric. For instance, if we consider MAE as evaluation criterion, CL_GMIFS performs best in simulation study 1 and in simulation study 2 with smaller and unequal class sizes; CR_L1 performs best in simulation study 2 with equal class sizes, in the scenario with unequal class sizes when the proportional odds assumption is violated, and in the CD study; CR_L1_path performs best in simulation study 3; and both CR_L1_path and OF perform best in the breast cancer study. Clearly, based on MAE, no single individual method consistently outperforms the others across all datasets. Interestingly, while CR_L1 is the top performing individual method in simulation study 2 across all four evaluation criteria both with equal class sizes and with unequal class sizes under proportional odds violation, its performance in simulation study 1 is relatively weaker than that of most other individual methods across the same metrics. This further emphasizes the pattern of no clear winner among the individual methods based on their performances across all the datasets. Conversely, the ensemble method delivers consistently strong and balanced results across datasets and evaluation metrics, making it a reliable and robust choice when the best‐performing individual method is unknown.

For the breast cancer study testing data, pairwise Kendall's rank correlations among individual models range from moderate to high values, indicating meaningful but not excessive dependence (Table S3). In this case, the ensemble method achieves the lowest MAE and the highest $\tau_{b}$ and accuracy, suggesting sufficient diversity among models enhances performance. In contrast, for the CD study, model predictions are highly correlated in the testing data, reflecting limited diversity (Table S4). Although the ensemble method remains competitive, improvements over individual models are relatively smaller than in the breast cancer study. Overall, while the ensemble method remains robust under high correlation, these results support the expectation that its relative advantage is greatest when diversity exists among models. Furthermore, if the selected evaluation metrics produce identical rankings, this effectively reduces diversity, limiting the potential benefit of the ensemble method. Therefore, diversity in model rankings across evaluation metrics is desirable for maximizing ensemble performance.

Although our ensemble ordinal classifier has shown strong performances in classification applications, it has a few limitations. First, training multiple individual methods and performing bootstrap aggregations can be computationally intensive, particularly for high‐dimensional datasets. Second, like any other ensemble method, this method relies greatly on the quality of its constituent individual methods, highlighting the importance of their careful selection. Third, the current implementation includes only ordinal classification approaches with established and publicly available R packages, excluding methods developed in other languages (e.g., Python), such as deep learning–based cumulative link neural networks [34], and methods incorporating unimodal regularization [35]. Fourth, two or more candidate models may have identical values for an evaluation metric. In such cases, the tied models are assigned ranks at random before performing the rank aggregation. Because the ensemble results are obtained by averaging across many bootstrap iterations, the effect of any single random tie‐breaking is minimal. Nevertheless, a dedicated tie‐breaking criterion is not implemented. Additionally, since final predictions are obtained via majority voting across bootstrap iterations, ties in voting are theoretically possible. Although such ties are expected to be rare given the large number of iterations, we did not implement a specific tie‐breaking procedure. Fifth, when one or more classes are rare in the overall population, a bootstrap or OOB sample may not include all classes, which can affect model evaluation and selection. Alternative strategies, such as stratified or weighted bootstrap sampling with appropriate reweighting during aggregation, could mitigate this issue. Addressing these limitations presents opportunities for future research to enhance the method's efficiency, scalability, and robustness.

In conclusion, the ensemble classifier consistently ranked among the top‐performing models, making it a reliable option for ordinal classification in high‐dimensional data. Its ability to integrate diverse individual classification methods and deliver balanced results makes it a valuable approach for complex classification problems, especially in medicine and public health.

## Funding

The authors have nothing to report.

## Ethics Statement

The authors have nothing to report.

## Consent

The authors have nothing to report.

## Conflicts of Interest

The authors declare no conflicts of interest.

## Supporting information


**Figure S1:** Distribution of the performances of the ensemble method and the individual algorithms across the evaluation metrics over the 100 Monte‐Carlo iterations, when all predictors are associated with the ordinal outcome and are correlated among themselves. CR_L1: Continuation ratio model with L_1 norm penalization; CR_L1_path: Continuation ratio model with L_1 norm penalization and path‐wise approach; CL_L1: Cumulative logit model with L_1 norm penalization; CL_GMIFS: Cumulative logit model with generalized monotone incremental forward stagewise penalization; OF: Ordinal forest.
**Figure S2:** Distribution of the performances of the ensemble method and the individual algorithms across the evaluation metrics over the 100 Monte‐Carlo iterations, when only the first 10 predictors are associated with the ordinal outcome. All predictors are correlated among themselves. CR_L1: Continuation ratio model with L_1 norm penalization; CR_L1_path: Continuation ratio model with L_1 norm penalization and path‐wise approach; CL_L1: Cumulative logit model with L_1 norm penalization; CL_GMIFS: Cumulative logit model with generalized monotone incremental forward stagewise penalization; OF: Ordinal forest.
**Figure S3:** Distribution of the performances of the ensemble method and the individual algorithms across the evaluation metrics over the 100 Monte‐Carlo iterations, when only the first 10 predictors are associated with the ordinal outcome. All predictors are uncorrelated among themselves. CR_L1: Continuation ratio model with L_1 norm penalization; CR_L1_path: Continuation ratio model with L_1 norm penalization and path‐wise approach; CL_L1: Cumulative logit model with L_1 norm penalization; CL_GMIFS: Cumulative logit model with generalized monotone incremental forward stagewise penalization; OF: Ordinal forest.
**Table S1:** Performance of the ensemble method and individual algorithms when only the first 10 predictors are associated with the ordinal outcome and class sample sizes are smaller and unequal. All predictors are correlated among themselves. Bold values indicate the best performance for each metric. Interquartile ranges (IQRs), based on 100 Monte‐Carlo iterations, are reported in parentheses. CR_L1: Continuation ratio model with $L_{1}$ norm penalization; CR_L1_path: Continuation ratio model with $L_{1}$ norm penalization and path‐wise approach; CL_L1: Cumulative logit model with $L_{1}$ norm penalization; CL_GMIFS: Cumulative logit model with generalized monotone incremental forward stagewise penalization; OF: Ordinal forest.
**Table S2:** Performance of the ensemble method and individual algorithms when only the first 10 predictors are associated with the ordinal outcome, with smaller and unequal class sample sizes and deviations from the proportional odds assumption. All predictors are correlated. Bold values indicate the best performance for each metric. Interquartile ranges (IQRs), based on 100 Monte‐Carlo iterations, are reported in parentheses. CR_L1: Continuation ratio model with $L_{1}$ norm penalization; CR_L1_path: Continuation ratio model with $L_{1}$ norm penalization and path‐wise approach; CL_L1: Cumulative logit model with $L_{1}$ norm penalization; CL_GMIFS: Cumulative logit model with generalized monotone incremental forward stagewise penalization; OF: Ordinal forest.
**Table S3:** Pairwise Kendall's Tau‐b values among the individual models in breast cancer study testing data. CR_L1: Continuation ratio model with L_1 norm penalization; CR_L1_path: Continuation ratio model with L_1 norm penalization and path‐wise approach; CL_L1: Cumulative logit model with L_1 norm penalization; CL_GMIFS: Cumulative logit model with generalized monotone incremental forward stagewise penalization; OF: Ordinal forest.
**Table S4:** Pairwise Kendall's Tau‐b values among the individual models in Crohn's disease study testing data. CR_L1: Continuation ratio model with L_1 norm penalization; CR_L1_path: Continuation ratio model with L_1 norm penalization and path‐wise approach; CL_L1: Cumulative logit model with L_1 norm penalization; CL_GMIFS: Cumulative logit model with generalized monotone incremental forward stagewise penalization; OF: Ordinal forest.

## Data Availability

The data that support the findings of this study are available in Gene Expression Omnibus database at https://www.ncbi.nlm.nih.gov/gds. These data were derived from the following resources available in the public domain: GSE22216: https://www.ncbi.nlm.nih.gov/geo/query/acc.cgi?acc=GSE22216; GSE3365: https://www.ncbi.nlm.nih.gov/geo/query/acc.cgi?acc=GSE3365.
