# Supplementary material for: An Ensemble Classifier for Ordinal Outcomes in High‐Dimensional Genomics Data
Source: Pharm Stat. 2026 May 10;25:e70097. doi: 10.1002/pst.70097 (PMC13158174; doi:10.1002/pst.70097)
Supplement: Supplementary file 1 — Figure S1: Distribution of the performances of the ensemble method and the individual algorithms across the evaluation metrics over the 100 Monte‐Carlo iterations, when all predictors are associated with the ordinal outcome and are correlated among themselves. CR_L1: Continuation ratio model with L_1 norm penalization; CR_L1_path: Continuation ratio model with L_1 norm penalization and path‐wise approach; CL_L1: Cumulative logit model with L_1 norm penalization; CL_GMIFS: Cumulative logit model with generalized monotone incremental forward stagewise penalization; OF: Ordinal forest. Figure S2: Distribution of the performances of the ensemble method and the individual algorithms across the evaluation metrics over the 100 Monte‐Carlo iterations, when only the first 10 predictors are associated with the ordinal outcome. All predictors are correlated among themselves. CR_L1: Continuation ratio model with L_1 norm penalization; CR_L1_path: Continuation ratio model with L_1 norm penalization and path‐wise approach; CL_L1: Cumulative logit model with L_1 norm penalization; CL_GMIFS: Cumulative logit model with generalized monotone incremental forward stagewise penalization; OF: Ordinal forest. Figure S3: Distribution of the performances of the ensemble method and the individual algorithms across the evaluation metrics over the 100 Monte‐Carlo iterations, when only the first 10 predictors are associated with the ordinal outcome. All predictors are uncorrelated among themselves. CR_L1: Continuation ratio model with L_1 norm penalization; CR_L1_path: Continuation ratio model with L_1 norm penalization and path‐wise approach; CL_L1: Cumulative logit model with L_1 norm penalization; CL_GMIFS: Cumulative logit model with generalized monotone incremental forward stagewise penalization; OF: Ordinal forest. Table S1: Performance of the ensemble method and individual algorithms when only the first 10 predictors are associated with the ordinal outcome and class sample sizes are smal [file PST-25-0-s001.docx]

**Supplementary Figure 1**

Distribution of the performances of the ensemble method and the individual algorithms across the evaluation metrics over the 100 Monte-Carlo iterations, when all predictors are associated with the ordinal outcome and are correlated among themselves. CR_L1: Continuation ratio model with L_1 norm penalization; CR_L1_path: Continuation ratio model with L_1 norm penalization and path-wise approach; CL_L1: Cumulative logit model with L_1 norm penalization; CL_GMIFS: Cumulative logit model with generalized monotone incremental forward stagewise penalization; OF: Ordinal forest.


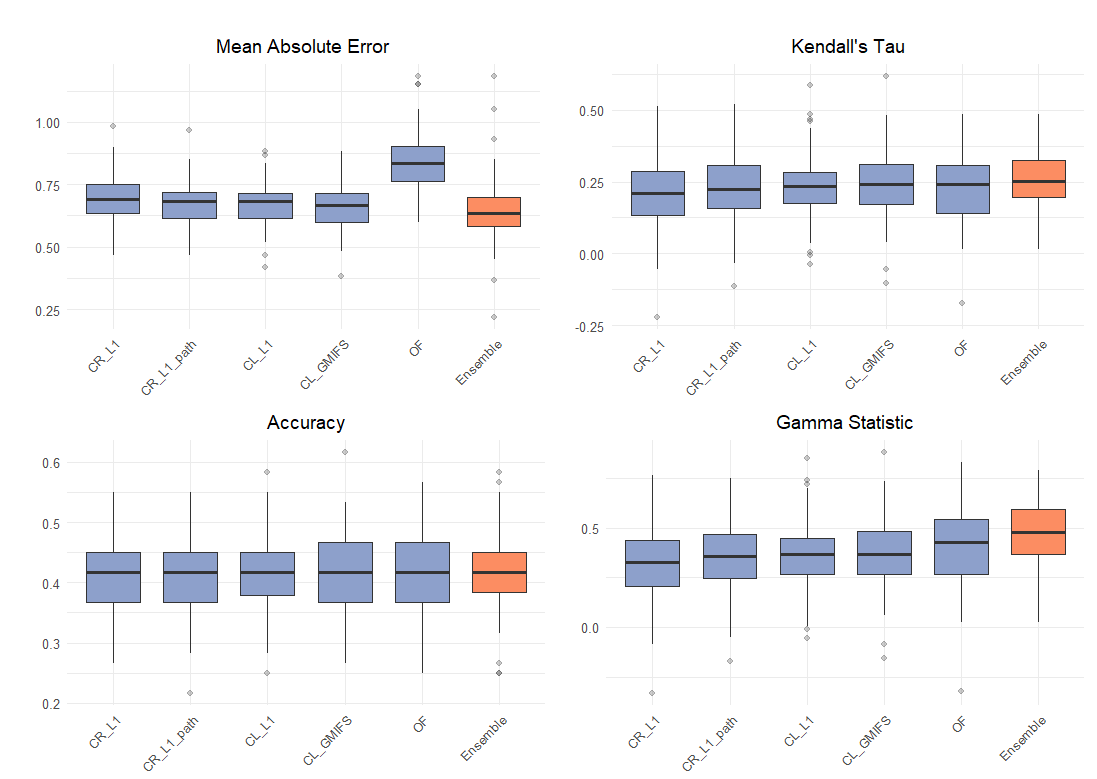


**Supplementary Figure 2**

Distribution of the performances of the ensemble method and the individual algorithms across the evaluation metrics over the 100 Monte-Carlo iterations, when only the first ten predictors are associated with the ordinal outcome. All predictors are correlated among themselves. CR_L1: Continuation ratio model with L_1 norm penalization; CR_L1_path: Continuation ratio model with L_1 norm penalization and path-wise approach; CL_L1: Cumulative logit model with L_1 norm penalization; CL_GMIFS: Cumulative logit model with generalized monotone incremental forward stagewise penalization; OF: Ordinal forest.


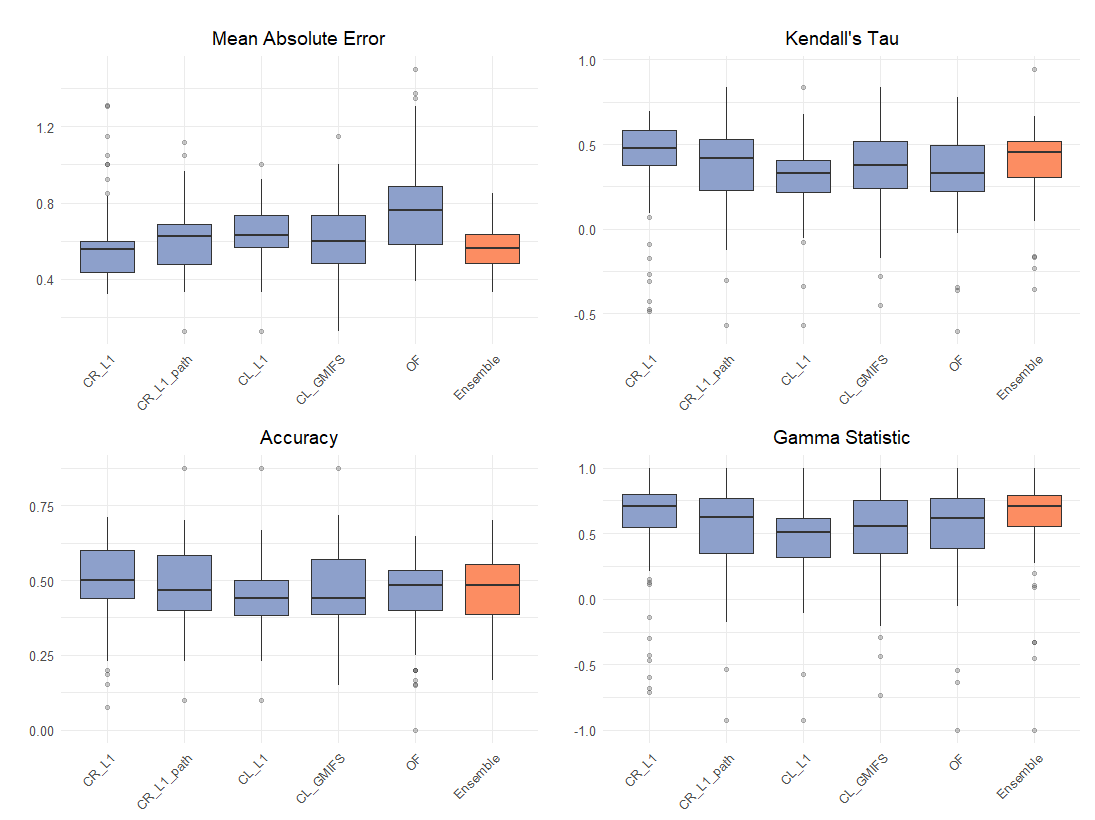


**Supplementary Figure 3**

Distribution of the performances of the ensemble method and the individual algorithms across the evaluation metrics over the 100 Monte-Carlo iterations, when only the first ten predictors are associated with the ordinal outcome. All predictors are uncorrelated among themselves. CR_L1: Continuation ratio model with L_1 norm penalization; CR_L1_path: Continuation ratio model with L_1 norm penalization and path-wise approach; CL_L1: Cumulative logit model with L_1 norm penalization; CL_GMIFS: Cumulative logit model with generalized monotone incremental forward stagewise penalization; OF: Ordinal forest.


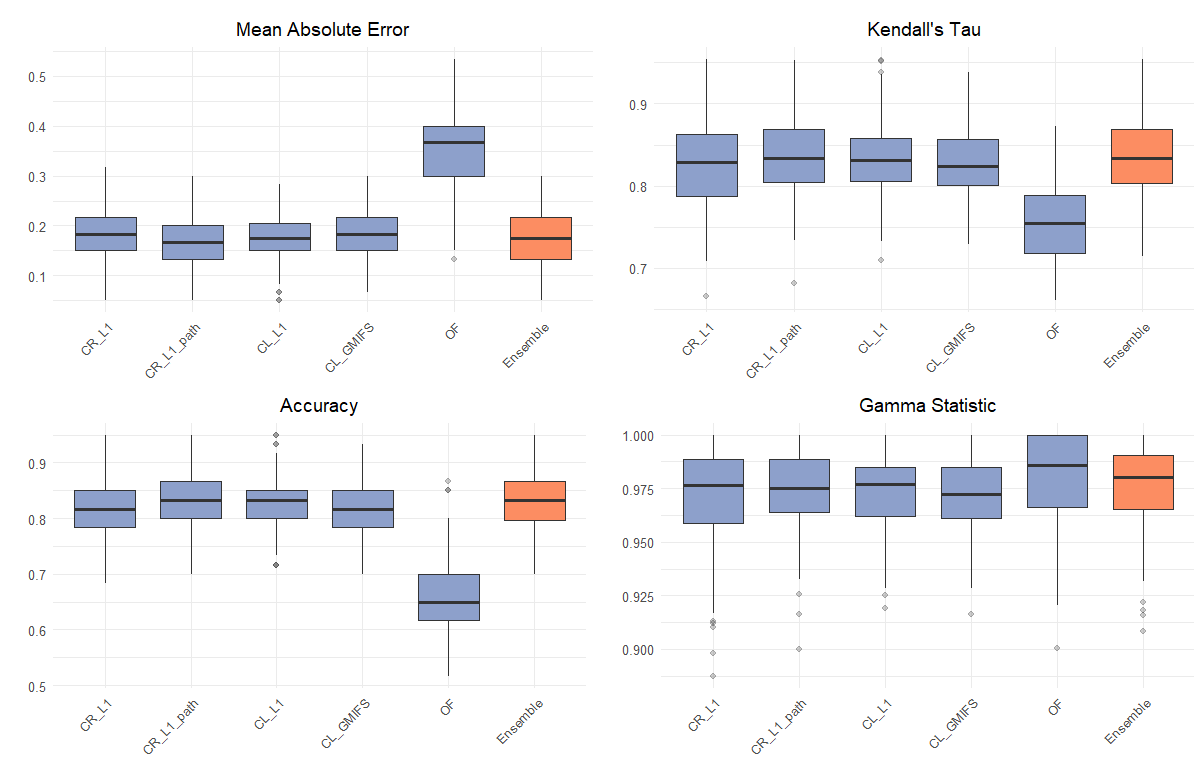


**Supplementary Table 1**

Performance of the ensemble method and individual algorithms when only the first ten predictors are associated with the ordinal outcome and class sample sizes are smaller and unequal. All predictors are correlated among themselves. Interquartile ranges (IQRs), based on 100 Monte-Carlo iterations, are reported in parentheses. CR_L1: Continuation ratio model with $L_{1}$ norm penalization; CR_L1_path: Continuation ratio model with $L_{1}$ norm penalization and path-wise approach; CL_L1: Cumulative logit model with $L_{1}$ norm penalization; CL_GMIFS: Cumulative logit model with generalized monotone incremental forward stagewise penalization; OF: Ordinal forest.

| **Model** | **MAE** | $\boldsymbol{\tau}_{\boldsymbol{b}}$ | **Accuracy** | $\boldsymbol{\gamma}$ |
| --- | --- | --- | --- | --- |
| CR_L1 | 0.6685 (0.3480) | 0.3490 (0.3696 | **0.5675** (0.1679) | 0.5763 (0.5500) |
| CR_L1_path | 0.6936 (0.2577) | 0.3105 (0.2505) | 0.4076 (0.1571) | 0.4900 (0.4001) |
| CL_L1 | 0.6835 (0.1538) | 0.3251 (0.2818) | 0.4126 (0.0979) | 0.4986 (0.3725) |
| CL_GMIFS | **0.6398** (0.2644) | 0.3583 (0.2766) | 0.4969 (0.1896) | 0.5273 (0.3964) |
| OF | 0.8170 (0.2539) | 0.2643 (0.1940) | 0.4984 (0.1274) | 0.5527 (0.6385) |
| Ensemble | 0.6444 (0.3435) | **0.3762** (0.2987) | 0.5454 (0.1392) | **0.6632** (0.5000) |

**Supplementary Table 2**

Performance of the ensemble method and individual algorithms when only the first ten predictors are associated with the ordinal outcome, with smaller and unequal class sample sizes and deviations from the proportional odds assumption. All predictors are correlated. Interquartile ranges (IQRs), based on 100 Monte-Carlo iterations, are reported in parentheses. CR_L1: Continuation ratio model with $L_{1}$ norm penalization; CR_L1_path: Continuation ratio model with $L_{1}$ norm penalization and path-wise approach; CL_L1: Cumulative logit model with $L_{1}$ norm penalization; CL_GMIFS: Cumulative logit model with generalized monotone incremental forward stagewise penalization; OF: Ordinal forest.

| **Model** | **MAE** | $\boldsymbol{\tau}_{\boldsymbol{b}}$ | **Accuracy** | $\boldsymbol{\gamma}$ |
| --- | --- | --- | --- | --- |
| CR_L1 | 0.6565 (0.3244) | 0.3744 (0.2659) | 0.6263 (0.1417) | 0.6145 (0.3095) |
| CR_L1_path | 0.6986 (0.2069) | 0.2868 (0.2402) | 0.5016 (0.2971) | 0.4498 (0.3743) |
| CL_L1 | 0.7529 (0.1250) | 0.2452 (0.1911) | 0.4503 (0.2265) | 0.3965 (0.2500) |
| CL_GMIFS | 0.6712 (0.2236) | 0.3371 (0.3209) | 0.5693 (0.2469) | 0.5319 (0.4347) |
| OF | 0.7444 (0.446) | 0.2633 (0.3795) | 0.5725 (0.2530) | 0.4265 (0.5829) |
| Ensemble | **0.6255** (0.3317) | **0.3910** (0.3620) | **0.6364** (0.1430) | **0.6225** (0.3491) |

**Supplementary Table 3**

Pairwise Kendall’s Tau-b values among the individual models in breast cancer study testing data. CR_L1: Continuation ratio model with L_1 norm penalization; CR_L1_path: Continuation ratio model with L_1 norm penalization and path-wise approach; CL_L1: Cumulative logit model with L_1 norm penalization; CL_GMIFS: Cumulative logit model with generalized monotone incremental forward stagewise penalization; OF: Ordinal forest.

|  | CR_L1 | CR_L1_path | CL_L1 | CL_GMIFS | OF |
| --- | --- | --- | --- | --- | --- |
| CR_L1 | 1 | 0.3602 | 0.3410 | 0.7176 | 0.4600 |
| CR_L1_path |  | 1 | 0.8800 | 0.4854 | 0.5976 |
| CL_L1 |  |  | 1 | 0.4589 | 0.6740 |
| CL_GMIFS |  |  |  | 1 | 0.5512 |
| OF |  |  |  |  | 1 |

**Supplementary Table 4**

Pairwise Kendall’s Tau-b values among the individual models in Crohn’s disease study testing data. CR_L1: Continuation ratio model with L_1 norm penalization; CR_L1_path: Continuation ratio model with L_1 norm penalization and path-wise approach; CL_L1: Cumulative logit model with L_1 norm penalization; CL_GMIFS: Cumulative logit model with generalized monotone incremental forward stagewise penalization; OF: Ordinal forest.

|  | CR_L1 | CR_L1_path | CL_L1 | CL_GMIFS | OF |
| --- | --- | --- | --- | --- | --- |
| CR_L1 | 1 | 0.9601 | 0.7384 | 0.8754 | 0.9250 |
| CR_L1_path |  | 1 | 0.7441 | 0.9083 | 0.9688 |
| CL_L1 |  |  | 1 | 0.8034 | 0.7266 |
| CL_GMIFS |  |  |  | 1 | 0.8891 |
| OF |  |  |  |  | 1 |
